# Bioactive Compounds and Antioxidant Activities in Different Fractions of Mango Fruits (*Mangifera indica* L., Cultivar Tommy Atkins and Keitt)

**DOI:** 10.3390/antiox11030484

**Published:** 2022-02-28

**Authors:** Marcello Salvatore Lenucci, Riccardo Tornese, Giovanni Mita, Miriana Durante

**Affiliations:** 1 Department of Biological and Environmental Sciences and Technologies (DiSTeBA), University of Salento, Prov.le Lecce-Monteroni, 73100 Lecce, Italy; marcello.lenucci@unisalento.it (M.S.L.); riccardo.tornese1@studenti.unisalento.it (R.T.); 2 Institute of Sciences of Food Production (ISPA), National Research Council (CNR), Prov.le Lecce-Monteroni, 73100 Lecce, Italy; giovanni.mita@ispa.cnr.it

**Keywords:** antioxidant activity, ascorbic acid, carotenoids, fatty acids, lupeol, mangiferin, phytosterols, pentacyclic triterpenes, phenolics, tocopherols

## Abstract

This study aims to describe and compare the distribution of bioactive compounds, the fatty acids profiles, and the TEAC hydrophilic and lipophilic antioxidant activities in different fruit fractions (pulp, peel, and kernel) of two mango cultivars (Tommy Atkins and Keitt). All fractions are sources of health-promoting bioactive compounds. Regardless of cultivars, pulp had the highest content of phytosterols (~150 mg/100 g dw), peels ranked first for pentaciclic triterpenes (from 14.2 to 17.7 mg/100 g dw), tocopherols, carotenoids, and chlorophylls, and kernels for phenolic compounds (from 421.6 to 1464.8 mg/100 g dw), flavonoids, condensed tannins, as well as hydrophilic and lipophilic antioxidant activities. Differences between the two cultivars were evidenced for ascorbic acid, which showed the highest levels in the peels and kernels of Keitt and Tommy Atkins fruits, respectively. Similarly, the concentration of dehydroascorbic acid was higher in the pulp of Tommy Atkins than Keitt. The highest percentage of saturated fatty acids was observed in pulp (~42%) and kernels (~50%), monounsaturated fatty acids in kernels (up to 41%), and polyunsaturated fatty acids in peels (up to 52%). Our results add information to the current knowledge on nutraceuticals’ distribution in different fractions of mango fruit, supporting its consumption as a healthy fruit and suggesting the great potential value of peels and kernels as sources of novel ingredients. Indeed, mango by-products generated during agronomic-to-industrial processing not only causes a significant environmental impact, but economic losses too. In this scenario, boosting research on conventional recovery methods offers eco-friendly solutions. However, green, novel biorefinery technologies may offer eco-friendly and profitable solutions, allowing the recovery of several more profitable by-products, sustaining their continuous growth since many bioactive compounds can be recovered from mango by-products that are potentially useful in the design of innovative nutraceutical, cosmeceutical, and pharmaceutical formulations.

## 1. Introduction

Mango (*Mangifera indica* L., Family Anacardiaceae) is one of the most popular tropical fruits of the 21th century thanks to its unique pleasant taste, aroma, and excellent nutritional value. Currently, fresh mango fruits are distributed to the worldwide market throughout the year. Indeed, different producing countries offer harvests at different periods, and standardized protocols for postharvest handling, warehousing, and transportation logistics are also available [1].

The world production of mangoes was estimated to be over 51 million tons in 2019, covering an area of more than 5 million hectares with up to 793 recognized cultivars [2]. Commercial production is reported in more than 87 countries, with India (20.5 million tons), China (5.2 million tons), Thailand (3.7 million tons), Mexico (2.4 million tons), Indonesia (2.3 million tons), and Pakistan (1.7 million tons) being within the top ten [1,3,4].

**Figure 1:**
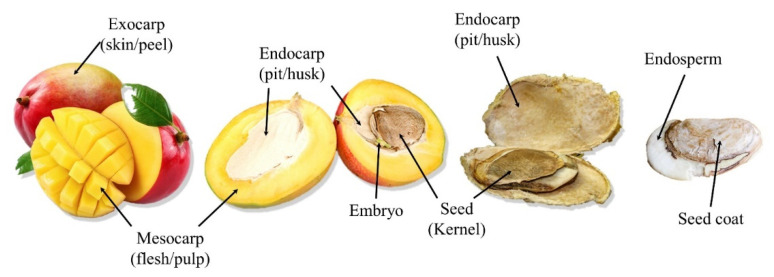
Anatomy of a typical mango fruit (*Mangifera indica* L.).

Anatomically, the fruit is classified as a deliquescent drupe (Figure 1) constituted by a thick, smooth, and glandular exocarp (peel/skin), green when unripe but turning golden yellow, orange-red, or crimson red at full ripeness, a fibrous, juicy edible mesocarp (flesh/pulp) orange-yellow in color, and a hard lignified endocarp (pit/husk) covering the single seed (kernel). Mango peels, pulp, and stone (pit plus kernel) account for 15–20%, 45–65%, and 20–45% of the whole fruit fresh weight, respectively depending mainly on genotype, with kernels contributing 45–75% of the entire stone weight [5,6].

Genotype also significantly affects the levels of several health-promoting compounds characterizing the pulp, peels, and kernel of mango fruit, whose variability is further enhanced by several interacting factors, including pedoclimatic conditions, cultural practices, ripening stage at harvest, as well as pre- and post-harvest treatments [7]. Indeed, mango fruit has been reported to exert nutritional, antioxidant, anti-inflammatory, metabolic, and immunomodulatory functions relevant to human health and well-being. The pulp, usually consumed fresh or processed into juice, puree, canned slices or diced, jam, pickles, and chutney, contains carbohydrates, proteins, organic acids, dietary fibers, and several bioactive secondary metabolites, including carotenoids, phenolic acids, and phytosterols. Besides, mango fruits are a source of monosaccharides (fructose, glucose, and sucrose) with varying levels between cultivars and ripening stages [8]. Nevertheless, due to the high amount of fructose, mangoes have been listed among the foods possibly increasing the symptoms of irritable bowel syndrome, a chronic gastrointestinal disorder [9]. The role of mango pulp phytocomplex in counteracting the production of reactive oxygen species (ROS) and pro-inflammatory mediators associated to cancer, cardiovascular, and neurodegenerative pathologies has been highlighted by several in vitro and in vivo studies [10]. Recently, Mirza et al. [11] summarized a comprehensive and critical evaluation of the cancer preventive and anticancer therapeutic potential of mango bioactives with a special focus on the cellular and molecular mechanisms of action. Furthermore, the prebiotic effects of mango polyphenols and dietary fiber and their potential in preventing gut microbiome dysbiosis have been demonstrated [12].

Peels and stones are by-products from the mango processing industry, wholly discarded as waste [13,14]. Given their interesting biochemical profiles, the exploitation of mango byproducts as sources of high-value functional ingredients for food supplementation and the formulation of pharmaceutical, nutraceutical, and cosmeceutical products has been repeatedly proposed, becoming an important aspect in waste management to improve agri-food production sustainability [11,12]. Thus, mango peel flour has been used to improve the nutritional quality and antioxidant properties of pasta, noodles, bread, sponge cakes, and biscuits [13,14,15], while phenolic-rich peel extracts revealed potent antiproliferative effects against different lines of human cancer cells [16]. Besides, the physicochemical characteristics of mango kernel fats, very similar to those of cocoa (*Theobroma cacao* L.), shorea (*Shorea* spp. Roxb. ex C.F.Gaertn.), karitè (*Vitellaria paradoxa* C.F.Gaertn.), and kokum (*Garcinia indica* Choisy) seed butters, makes it an interesting alternative for cosmetic applications, being perfect for moisturizing skin and hair. In addition, it has a subtle, almost non-existent aroma and is loaded with vitamins A and E, antioxidants, and essential fatty acids, making it an optimal choice for scented recipes, as well as for pharmaceutical and food purposes [17,18]. Recently, Kaur et al. [19] found that cocoa butter could be replaced up to 80% by mango butter in the preparation of dark chocolate, showing excellent acceptability and, simultaneously, ensuring the reduction of environmental pollution caused by mango by-products, also benefitting the processing unit’s operation economy.

Among the huge number of cultivated mango varieties, only a few are widely exported, the most popular being Kent, Tommy Atkins, Haden, and Keitt for their firmer fruits well suited to long-distance transportation [20]. As commonly observed for most plant organs, the phytochemical composition and nutritional value of mango fruits are affected by several factors, with their distinct anatomical fractions usually showing quantitative and/or qualitative peculiarities somehow related to the tissue specific function. Thus, this study aims at evaluating the variability in the soluble and insoluble phenolics, flavonoids, condensed tannins, ascorbic and dehydroascorbic acids, phytosterols, pentacyclic triterpenes, tocopherols, carotenoids, chlorophylls, fatty acids, and antioxidant activities of different fruit tissues (pulp, peels, and kernels) from two mango (Tommy Atkins and Keitt) cultivars imported in Italy from Brazil. These cultivars were selected as they are the most commonly sold mangoes in Italy and are easily available all year round on the market.

## 2. Materials and Methods

### 2.1. Chemicals and Reagents

All HPLC grade solvents and reagents and most high purity standards for qualitative/quantitative determinations were purchased from Sigma–Aldrich (Milan, Italy). Standards of carotenoids and chlorophylls were obtained from Cayman chemicals (Ann Arbor, MI, USA) and DHIWater & Environment (Copenhagen, Denmark), respectively.

### 2.2. Plant Material Sampling and Preparation

Ten commercially ripe mango fruits, similar in size and caliber, with no visible mechanical damage and/or pathogen/pest injury, were sampled for each of the two investigated cultivars (Tommy Atkins and Keitt) in a local market (MD Discount, Lecce, Italy), in five different rounds in the period April–June 2020. Both cultivars were imported from Brazil and distributed in Italy by “Belleza” (Torres Tropical Fresh, Barendrecht, Holland). Each sampling round represented one of the five independent biological replicas.

**Figure 2:**
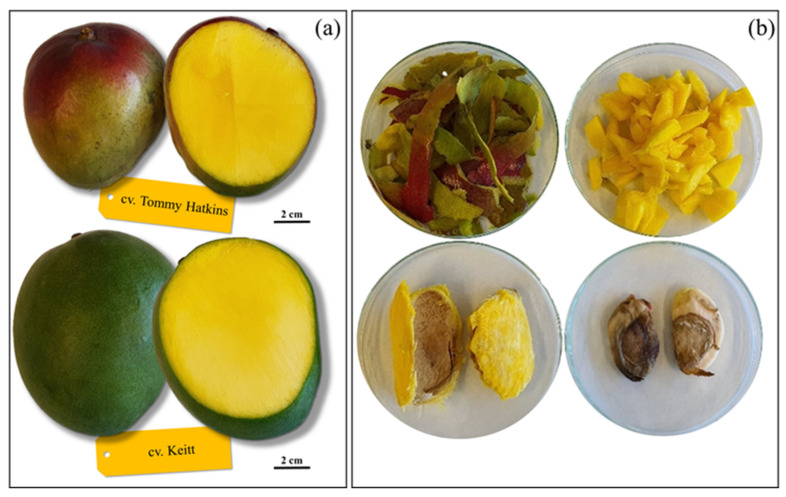
(**a**) External appearance and longitudinal sections of ripe fruits of Tommy Atkins and Keitt cultivars. (**b**) Macroscopic appearance of the peels, pulp and kernels isolated from representative fruits (Tommy Atkins cultivar).

All fruits were manually washed, visually inspected for peel and pulp color, and assayed for flesh firmness and total soluble solids for a proper classification of their ripening class according to the USA National Mango Board Guidelines (www.mango.org, accessed on 20 December 2021). At least three fruits, uniform for ripening, were selected from each of the sampling rounds and carefully peeled with a stainless steel potato slicer (peel thickness about 1.5 mm). Pulp was finely chopped, and the kernel was recovered by splitting open the hard pit (Figure 2). Peels, pulp, and kernels were immediately frozen in liquid nitrogen, dehydrated to constant weight by a Christ ALPHA 2–4 LSC freeze-dryer (Martin Christ Gefriertrocknungsanlagen GmbH, Osterode am Harz, Germany), and ground in a laboratory mill (ZM200, Retsch GmbH, Hannoversch Münden, Germany) through a 1-mm sieve. The obta Hannoversch Mündenined dehydrated powders were stored in vacuum-sealed polyethylene bags covered with aluminum foil at −80 °C until analysis.

### 2.3. Determination of Mango Fruit Flesh Firmness and Total Soluble Solids

Flesh firmness was measured with a mechanical fruit pressure tester with an 8-mm probe (model FT 327, Facchini S.r.l., Alfonsine, Ravenna, Italy). After peel removal, measurements were taken at the two opposite sides of the fruit equatorial plane. Total soluble solids, expressed as °Brix, were determined at room temperature using a digital refractometer (DBR95 Giorgio Bormac S.r.l., Carpi, Modena, Italy) on the fresh juice obtained by squeezing the mango pulp.

### 2.4. Extraction and Determination of Soluble and Insoluble-Bound Phenolic Compounds

Extraction of phenolic compounds was carried out as described by Ferreres et al. [21] with slight modifications. Briefly, soluble phenolics (free plus conjugated) were extracted from triplicate aliquots (0.1 g dry weight, dw) of each powdered sample with 5 mL methanol (80% *v*/*v*) in a Labsonic177 LBS1-10 ultrasonic bath (Falc Instruments, Treviglio, Italy) for 30 min, followed by centrifugation at 4000× *g* for 15 min in an Avanti JXN-26 centrifuge (Beckman Coulter Ltd., High Wycombe, UK). The insoluble-bound phenolic compounds were released via alkaline hydrolysis (2 M NaOH, 4 h, in the dark at room temperature) of the residue (pellet) from the initial methanol (80% *v*/*v*) extraction, according to Laddommada et al. [22].

Soluble and insoluble-bound phenolic total contents were determined spectrophotometrically using the Folin–Ciocalteu method [23]. Briefly, each extract (50 µL) was mixed with 50 µL of the Folin–Ciocalteau phenol reagent and 450 µL distilled water. After 5 min, 500 µL of 7% Na_2_CO_3_ and 200 µL of distilled water were added. The mixture was left at room temperature for 90 min in the dark. Absorbance was read at 750 nm in a DU650 spectrophotometer (Beckman Coulter Ltd., High Wycombe, UK). The results were expressed as mg gallic acid equivalents (GAE)/100 g dw. The total phenolic content was calculated as the sum of soluble plus insoluble-bound fractions.

Besides, a qualitative–quantitative analysis of the major individual phenolics was performed by HPLC equipped with a photodiode array detector (DAD) (Agilent Tecnologies, Waldbronn, Germany) using a Phenomenex-luna 5 μm C18 (2) 100 Å column (250 × 4.6 mm), (Phenomenex, Torrance, CA, USA) as reported by Durante et al. [24]. Briefly, to obtain the soluble-conjugated forms, aliquots (500 µL) of each soluble phenolic extract were hydrolyzed adding an equal volume of 2.4 M methanolic HCl followed by incubation for 4 h in the dark at room temperature. The hydrolyzed samples were evaporated to dryness under a stream of nitrogen and resuspended in 500 µL methanol (80% *v*/*v*). The soluble conjugated forms were quantified by subtracting for each peak identified in the hydrolized extract the area of the eventual corresponding peak from the untreated extract. Mangiferin (C-glucosyl xanthone), rutin, and luteolin-7O-glucoside were directly quantified in the not hydrolysed extract based on the retention times of the respective authentic standards. The results were expressed as mg/100 g dw.

### 2.5. Extraction and Determination of Flavonoids and Condensed Tannins

Triplicate aliquots (0.3 g dw) of each powdered sample were extracted twice with 1.5 mL absolute methanol under vigorous shaking (300 rpm) for 16 h at 4 °C. The particulate was separated by centrifugation at 8800× *g* for 10 min and the extracts (supernatants) were used to assay the content of total flavonoids and condensed tannins according to Zhishen et al. [25] and Broadhurst and Jones [26], respectively. Briefly, for flavonoid determination, a mixture of the extract (50 µL), distilled water (450 µL), and 5% NaNO_2_ (30 µL) was incubated for 5 min at room temperature. Subsequently, 10% AlCl_3_ (60 µL) was added, followed, after further 6 min of incubation, by 1M NaOH (200 µL) and distilled water (210 µL). For condensed tannins, the extract (100 µL) was mixed with 4% vanillin-methanol solution (600 µL) and 12 M HCl (300 µL). The mixture was incubated at 20 °C in the dark for 10 min. The absorbance was read at 510 for total flavonoids and 500 nm for condensed tannins in a Beckman DU650 spectrophotometer. In both cases, the contents were expressed as mg catechin equivalents (CE)/100 g dw.

### 2.6. Extraction and Determination of Ascorbic Acid (AsA) and Dehydroascorbic Acid (DHA) Contents

AsA and DHA contents were determined as reported by Kampfenkel et al. [27] on 0.2 g dw triplicate aliquots of each powdered sample. AsA and DHA were extracted using 6% metaphosphoric acid (1 mL). The absorbance was read at 525 nm in a Beckman DU650 spectrophotometer. Results were expressed as mg/100 g dw.

### 2.7. Phytosterols and Pentacyclic Triterpenes Determination

Triplicate aliquots of the pulp (2 g), peel (0.5 g), and kernel (0.1 g) powders were extracted with 5 mL of *n*-hexane under mechanical stirring (300 rpm) at 4 °C for 16 h. After centrifugation at 6000× *g* for 5 min, the organic phase was evaporated to dryness under a stream of nitrogen and solubilized in n-hexane (1 mL). Extracts (100 µL) were analyzed for phytosterol and pentacyclic triterpene contents as reported by Durante et al. [28] using an Agilent 5977E Series GC/MS system equipped with a HP-5ms column (30 m, 0.25 mm i.d., 0.25 mm film thickness) (Agilent Technologies, Santa Clara, CA, USA).

### 2.8. Isoprenoids (Tocopherols, Carotenoids and Chlorophylls) Determination

The method of Fraser et al. [29] was used for isoprenoid extraction. Freeze dried samples (100 mg, triplicate aliquots for each independent replica) were suspended in 3 mL of methanol and 3.6 mL of water and extracted at 4 °C under vigorous shaking. At 10-min intervals, 3 mL of 50 mM Tris-HCl pH 7.5 (containing 1 M NaCl) were added followed by 8 mL anhydrous chloroform, continuing the extraction under the same conditions. After centrifugation (6000× *g*, 5 min at 4 °C), the organic upper phase was recovered and evaporated to dryness under a stream of nitrogen. The dried samples were suspended in 100 µL ethyl acetate and assayed, qualitatively–quantitatively as in Durante et al. [30] using an Agilent 1100 Series HPLC system. Absorbance was registered at 290 nm for tocopherols, 475 nm for carotenoids, and 675 nm for chlorophylls.

### 2.9. Assay of Hydrophilic and Lipophilic Antioxidant Activities

Hydrophilic and lipophilic antioxidants were sequentially extracted from 0.4 g dw of each sample (triplicate aliquots) with 1 mL of methanol (hydrosoluble antioxidants) and 1 mL of acetone (liposoluble antioxidants) under constant shaking (300 rpm) at room temperature in the dark for 1 h, according to Perez-Jimenez et al. [31]. The hydrophilic (HAA) and lipophilic (LAA) antioxidant activities were measured by the TEAC (trolox equivalent antioxidant capacity) assay as reported by Durante et al. [32]. The total antioxidant activity (TAA) was calculated as the sum of HAA and LAA. The results were expressed as mol trolox equivalents (TE)/100 g dw.

### 2.10. Fatty Acids Determination

Total lipids were extracted from triplicate aliquots of freeze dried mesocarp (0.2 g), exocarp (0.5 g), and kernel (0.1 g) with 5 mL of *n*-hexane under mechanical stirring (300 rpm) at 4 °C for 16 h. After centrifugation at 4500× *g* for 5 min, the organic phase was evaporated to dryness under a stream of nitrogen. A methanolic solution (3 mL) of 0.5 M NaOH was added to each extract. The mixture was incubated at 100 °C for 5 min in a water bath. After cooling, 2.0 mL of boron trifluoride-methanol solution (14% *w*/*v*) were added. The sample was incubated at 100 °C for 30 min in a water bath, then cooled before the addition of 1 mL of hexane and 1 mL of a 0.6% (*w*/*v*) sodium chloride solution. After centrifugation (6000× *g*, 2 min at 4 °C), the organic upper phase was recovered and analyzed by GC/MS analysis as described in Durante et al. [30] using an Agilent 5977E GC/MS system equipped with a DB-WAX column (60 m, 0.25 mm i.d., 0.25 mm film thickness) (Agilent Technologies, Santa Clara, CA, USA).

### 2.11. Statistical Analysis

Statistical analysis was based on a one-way ANOVA test. The Tukey post hoc method was applied to establish significant differences between means (*p* < 0.05). Correlations were calculated using Pearson’s correlation coefficient (r). Statistical comparisons were performed using SigmaStat software, version 14.0 (Systat Software Inc., Chicago, IL, USA). For a visual analysis of data, principal component analysis PCA was performed on the complete data matrix of each mango cultivar using the XLSTAT software (Addinsoft, Paris, France). The analysis was carried out by plotting the mean values of each evaluated parameter (variables) within pulp, peels, and kernels isolated from the fruits of each cultivar.

## 3. Results and Discussion

Being climacteric, mango fruits from tropical countries undergoing long-distance transportation (e.g., to the EU markets) are usually harvested firm at the mature, green stage and complete ripening off-vine [33]. Thus, large retail distribution typically offers a selection of mango fruits heterogeneous for ripening, with some at the ready-to-eat stage and others still unripe. Generally, at harvest, the total soluble solids of mango fruit have values between 7 and 9 °Brix, which increase during ripening up to 13–15 °Brix. Simultaneously, a reduction in pulp firmness and an intensification of the yellow color of the pulp occurs [34]. In the present research, mango fruits uniform for ripening were carefully selected based on the instrumental determination of pulp firmness and total soluble solids, and on the visual comparison of the pulp color with cultivar specific reference chromatic scales (www.mango.org; accessed on 15 April 2020). In particular, the Tommy Atkins fruits selected for this research had values of firmness and total soluble solids of 3.9 kg/cm^2^ and 12.8 °Brix, respectively, while in the cultivar Keitt the corresponding average values were 3.3 kg/cm^2^ and 11.5 °Brix.

### 3.1. Polyphenolic Composition

Polyphenols (including flavonoids, xanthones, and phenolic acids) are the most abundant dietary antioxidants of mango fruits. Well known for their ability to scavenge free radicals through hydrogen atom transfer, single electron transfer, and/or chelation of metal cations, polyphenols are thought to account for a large part of the biological and pharmacological activities attributed to mango fruits [35]. Plant polyphenols occur in soluble or insoluble forms, both with important nutritional value as they have a different metabolic fate and biological significance in humans [36]. Soluble phenolics generally segregate within the vacuole, free or conjugated to oligosaccharides and peptides through ester or ether bonds, and play a pivotal role in plant defense against ultraviolet radiation, aggression by pathogens, parasites, and predators, as well as in contributing to plant organ color. Instead, insoluble phenolics are covalently bound to the cell wall polymers and exert a mainly structural role [37].

**Figure 3:**
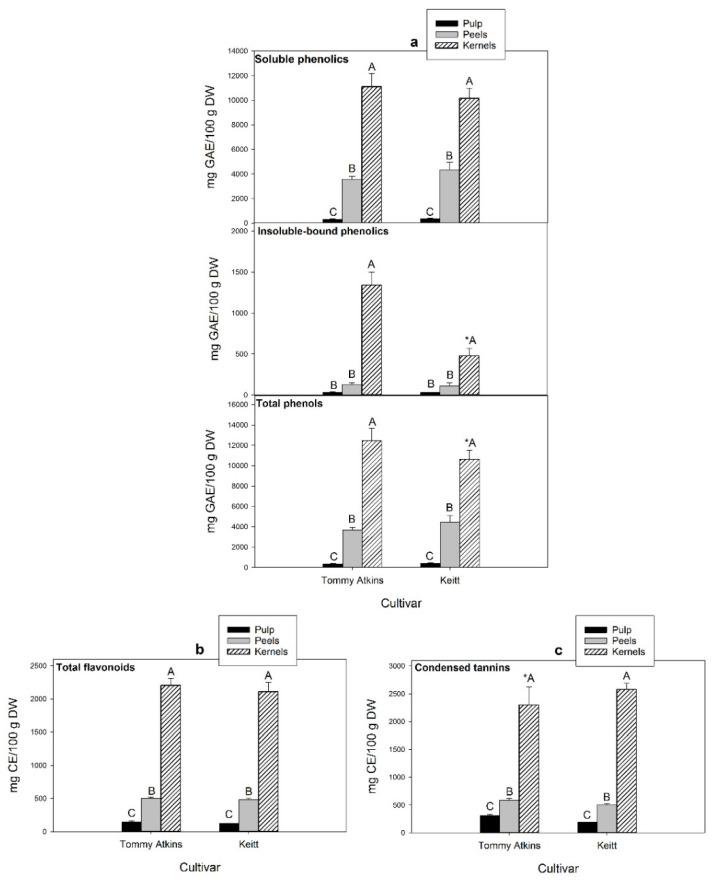
Contents of (**a**) soluble, insoluble-bounds and total phenolics; (**b**) total flavonoids and (**c**) condensed tannins in the pulp, peels and kernels of mango fruits (cvs Tommy Atkins and Keitt). Data, expressed as mg gallic acid equivalents (GAE)/100 g dw or mg catechin equivalents (CE)/100 g dw, are the mean ± standard deviation of five independent replicates (*n* = 5). Data were submitted to one-way analysis of variance (ANOVA), differences among groups were detected using multiple comparison procedures (Tukey post hoc test, *p* < 0.05). For each trait, significant differences between cultivars within each fruit fraction were highlighted by an asterisk (*), different capital letters indicate differences among different fraction for the same cultivar (*p* < 0.05).

The average contents of phenolic compounds (soluble, insoluble bound, and total), flavonoids, and condensed tannins in the pulp, peels, and kernels obtained from ripe mango fruits of the cultivars Tommy Atkins and Keitt are reported in Figure 3. Regardless of cultivar and fruit fractions, soluble phenolics largely exceeded the insoluble bound, representing up to 97% of the total. Significant differences were detected among fruit fractions (Figure 3a), with kernels showing the highest contents of both soluble and insoluble-bound phenolics, followed by peels and pulp. No statistically significant variation was detected between the two cultivars, except for the concentration of kernel insoluble bound phenolics, much higher in Tommy Atkins (1340 mg GAE/100 g dw) than in Kent (480 mg GAE/100 g dw). The present findings are consistent with those of other authors, who reported total phenolic concentrations in the range of 19,749–20,034, 2032–9200, and 253–699 mg GAE/100 g dw, respectively, in the kernels, peels and pulp from ripe fruits of the same cultivars [38,39,40,41].

Interestingly, large differences among pulp, peels, and kernels of both cultivars were found for the conjugated phenolic content, which contributed to 95–97%, 83–94%, and 11–29% of the total soluble phenolics, respectively (Table 1), possibly in relation to the metabolic state of the tissues. Free phenolics are usually associated to dead or dying tissues. Thus, the high content found in the kernels may relate to seed development, culminating in programmed cell death and hardening of tissues enclosing the embryo (e.g., seed coat). Besides, conjugation is of considerable biological significance. Indeed, the degree of glycosylation was reported to significantly affect the antioxidant activity of phenolics. Aglycones of quercetin and myricetin, for example, demonstrated higher antioxidant activity than their corresponding glycosides, though conjugated forms are better absorbed in humans [42].

Flavonoids represent a significant portion of soluble phenolics in ripe mangoes. Thus, their total content followed a similar distribution trend in the different fruit fractions (Figure 3b), being the highest in kernels (2110–2204 mg CE/100 g dw), followed by peels (480–504 mg CE/100 g dw) and pulp (119–147 mg CE/100 g dw), with no statistically significant differences between cultivars. A marked genotype-associated variability in the content of total phenolics and flavonoids (within the range 2930–6624 mg GAE/100 g dw and 502–795 mg CE/100 g dw, respectively) was instead reported by Marcillo-Parra et al. [43] in the peels of three mango cultivars (Tommy Atkins, Kent, and Haden) from the Ecuadorian region. Besides, compared to our results, lower and higher flavonoid levels were reported for the kernels (1300 mg CE/100 g dw) and peels (700 mg CE/100 g dw) of Keitt mangoes by Dorta et al. [44], indicating the existence of a certain intravarietal variability. This was further supported by Peng et al. [41], who found only 186 mg CE/100 g dw in the peels of the same cultivar.

Condensed tannins (proanthocyanidins) are oligo/polymeric flavonoids consisting of flavan-3-ol structural units (i.e., catechin, epicatechin, and/or epigallocatechin) naturally present in many vegetables, seeds, and fruits. Mango kernels also ranked first for this class of compounds (2299–2580 CE mg/100 g dw), followed by peels (500–584 CE mg/100 g dw) and pulp (190–313 mg CE/100 g dw) with no significant differences among cultivars, except for kernels (Figure 3c). High levels of condensed tannins (1323 mg CE/100 g dw) were previously reported by Makkar et al. [45] in mango kernels, which have proven to be among the agro industrial byproducts with the greatest potential as sources for the large scale extraction of these compounds. Given their natural antioxidant and antimicrobial activity, condensed tannins are commercially used as a preservative to stabilize food colors, prevent rancidity, and avoid microbial growth [46]. Levels of up to 7.0 and to 0.58 mg leucoanthocyanidin equivalents (LE)/100 g dw were reported, respectively, in mango kernels and peels of the cultivar Keitt by Hung et al. [38], who highlighted a large variability associated with the used solvent and extraction temperature and the need for optimizing the extraction parameters to obtain extracts rich in antioxidants from mango byproducts.

With regard to the profiles of the major phenolics (free and conjugated forms), overall, seven individual compounds were identified in the tree fruit fractions (Table 1).

Kernels of both cultivars were characterized by high levels of free methyl gallate (up to 2126.5 mg/100 g dw) and propyl gallate (about 1400 mg/100 g dw), also detected in the peels, though in considerably lower concentrations. Methyl gallate was the only free polyphenol identified in the pulp of both varieties, where it was present also in the conjugated form together with large amounts of gallic acid. Free gallic acid was detected only in the kernels with a significant difference in concentration between Tommy Atkins (14.6 mg/100 g dw) and Keitt (104.2 mg/100 g dw) cultivars. On the contrary, Kim et al. [47] reported free gallic acid as the major phenolic acid in mango pulp (cultivar Tommy Atkins).

Gallic acid and its methyl- and propyl-derivatives are known to exert strong antioxidative and antiviral effects in vitro and in vivo [48,49]. Besides, gallic acid and methyl gallate extracted from seeds of white catamaran tree (*Givotia rottleriformis* Griff. ex Wight) reduced the growth of human epidermoid carcinoma cells and exhibited inhibitory activity against hepatitis C virus [50,51,52].

Though identified in a number of other plants, mangiferin is a polyphenol of mango distinctive for its high levels characterizing the fruits from which it is mainly produced [53]. Mangiferin has been reported to possess a broad range of therapeutic effects, including anti-inflammation, anti-diabetic, immunomodulatory, anti-tumor, and antioxidant activities [54,55]. It has been used in many food supplements and is considered one of the main active constituents in more than 40 polyherbal formulations in traditional Chinese medicine [53]. Mangiferin was differentially distributed in the fruit of mango. It was the predominant soluble conjugated phenolic compound in Tommy Atkins peels (411 mg/100 g dw) but was not observed in Keitt peel samples. Mangiferin was also present, at lower and significantly different levels, in the kernels of the two cultivars. In agreement with our findings, Luo et al. [56] found the highest mangiferin level in the peels (4–749 mg/100 g dw), followed by kernels (14–243 mg/100 g dw) and pulp (0–20 mg/100 g dw) with a large intervarietal variability among the eleven Chinese mango cultivars assayed. Differently, López-Cobo et al. [57] found higher levels of mangiferin in the kernels (22–73 mg/100 g dw) than in peels (4–30 mg/100 g dw) and husk (2–17 mg/100 g dw) of three mango cultivars (Keitt, Osteen and Sensación) from Spain, while it was not detected in the pulp. A genotype-dependent variation of mangiferin levels was also reported by Berardini et al. [58] who found a high amount of mangiferin in Tommy Atkins peels (126.3 mg/100 g dw), but much lower levels in samples from Haden (1.1 mg/100 g dw) and Kent (1.4 mg/100 g dw) cultivars. Rutin was also detected in the peels (30.2–65.2 mg/100 g dw) and kernels (25.5–40.3 mg/100 g dw), while luteolin-7-*O*-glucoside was exclusive to the peels (5.3–13.1 mg/100 g dw) of both cultivars.

### 3.2. Ascorbic (AsA) and Dehydroascorbic (DHA) Acid Contents

**Figure 4:**
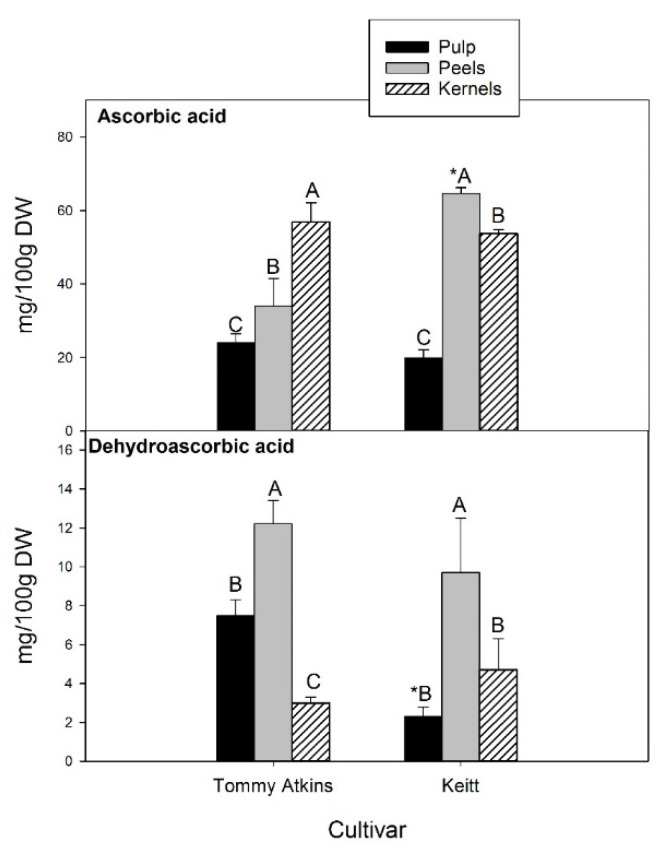
Ascorbic and dehydroascorbic acid content in three fractions (pulp, peel and kernel) of mango fruit. Data, expressed as mg/100 g dw, are the mean ± standard deviation of five independent replicates (*n* = 5). Data were submitted to one-way analysis of variance (ANOVA), differences among groups were detected using multiple comparison procedures (Tukey test). Significant differences between cultivars within each fruit fraction were highlighted by an asterisk (*); different capital letters indicate differences among fruit fractions for the same cultivar (*p* < 0.05).

Ascorbic acid (AsA) in plants plays a central role in several physiological processes, including cell redox potential buffering, regulation of photosynthesis and production of phytohormones, control of cell division and growth, and signal transduction. It is also a chief component of the efficient antioxidant machinery evolved by plants to counteract harmful reactive species [59]. With regard to human nutrition, AsA represents the dominant biologically active form of vitamin C present in most edible plants including mango, although the pulp of the ripe fruits is not considered a major source of this essential nutrient. Besides, its reversibly oxidized form, dehydroascorbic acid (DHA), has also been detected in conventional and organic mango fruits at much lower levels and reported to contribute up to 18% of the total Vitamin C, though large variations were observed depending on cultivar, fruit tissue, post-harvest manipulations/treatments, and storage [60,61]. An inverse correlation was found between mango peel browning resulting from chilling injuries and ascorbic acid concentrations [62]. AsA levels between 2.8- and 19.0-fold those of DHA, depending on fraction and cultivar, were observed in the present study (Figure 4).

In kernels, AsA and DHA contents were about 55.0 and 3.8 mg/100 g dw, respectively, with no significant differences between Tommy Atkins and Keitt, while showing a different ranking in the different fruit fractions. In Tommy Atkins, kernels ranked first for AsA content, followed by peels (34.0 mg/100 g dw) and pulp (24.0 mg/100 g dw). In the cultivar Keitt, the highest concentration was registered in the peels (64.6 mg/100 g dw), which also turned out to be the fruit fraction with the highest DHA content (on average 11.0 mg/100 g dw). There were no statistically significant differences between cultivars. With regard to pulp, Tommy Atkins showed a DHA level 3.3-fold higher than Keitt. Our values are consistent with those reported by Sogi et al. [40] and Carvalho et al. [63], who found ascorbic acid contents from 68.5 to 84.74 mg/100 g in dried peel powder and 61.2–74.5 mg/100 g dw in the kernels of market-ripe mangoes (cv Tommy Atkins).

### 3.3. Lipophilic Bioactives

A qualitative–quantitative determination of the main lipophilic phytochemicals of mango fruits (i.e., phytosterols, pentacyclic triterpenes, tocopherols, carotenoids, and chlorophylls) is reported in Table 2.

Phytosterols are the predominant lipophilic class of bioactives in all mango fruit tissues. They are well known for their cholesterol-lowering effects, anti-inflammatory and antioxidant properties, and the benefits they offer to the immune system. Previous analyses of three mango cultivars from Spain indicated peels as the fruit fraction richest in phytosterols with a total amount in the range 56.6–69.3 mg/100 g dw, followed by kernels (31.8–54.4 mg/100 g dw) and pulp (24.5–40.1 mg/100 g dw) [64]. However, our findings support a different ranking with pulp first (up to 151.5 mg/100 g dw), followed by kernels (106.9–135.5 mg/100 g dw) and peels (55.9–83.0 mg/100 g dw). Values still lower but much closer to those we found in the pulp were reported by Vilela et al. [65] comparing 12 cultivars from Madeira Island, though a large variability in the level of total phytosterols within a range of 34.3–103.0 mg/100 g dw was reported among genotypes. β-sitosterol was the only sterol identified in the kernels of both cultivars and contributed to over 80% and 70–80% of the total sterols in the pulp and kernels, respectively. Stigmasterol and campesterol were also detected, though with different relative percentages, in agreement with the reports of other authors [64,65,66].

Pentacyclic triterpenes exhibit a large range of biological activities, including anti-inflammatory, anti-cancer, and gastroprotective properties [67,68]. In this work, two pentacyclic triterpenes, α-amyrin and lupeol, were identified in the pulp and peel fractions of both cultivars, with peels showing the highest total concentration (17.7 and 14.2 mg/100 g dw in Tommy Atkins and Keitt, respectively) (Table 2). In all fruit fractions, α-amyrin and lupeol were almost equivalent in concentration, with the exception of the Tommy Atkins pulp, in which the α-amyrin level was about twice as much. Recently, Mannowetz et al. [69] reported that lupeol may find application as a non-hormonal contraceptive, inhibiting sperm hyperactivation critical to egg cell penetration of the zona pellucida. In accordance with the findings of Ruiz-Montañez et al. [70] and Jyotshna et al. [71], lupeol concentration was 1.7–4.0 times higher in peels than in pulp, indicating a potential use of this massive by-product of mango processing as a source for the extraction of this pharmacologically active triterpenoid.

Tocopherols are known as potent antioxidants with vitamin E activity and essential micronutrients in the human diet. In mango fruit, their accumulation is highly correlated with *p*-hydroxyphenyl pyruvate dioxygenase gene expression during ripening, which is in turn ethylene induced and differentially expressed depending on cultivars [72]. The highest tocopherol total content was registered in the peels of both cultivars, though with statistically significant differences between Tommy Atkins and Keitt (10.6 and 12.3 mg/100 g dw, respectively), while in pulp it was 3.3–4.6-fold lower. Mango kernels showed a very low amount of total tocopherols (0.5–0.2 mg/100 g dw) with no significant differences between cultivars. The α-isoform prevailed over the β in kernels (70% of the total tocopherols), and it was the only isoform identified in the pulp, in agreement with the data reported by López-Cobo [64]. In the kernels, however, the two isoforms were in almost equivalent concentrations. Differently, Jin et al. [66] reported that mango kernel fat was mostly dominated by α-tocopherol (>40%) and presented high percentages (15–45%) of the δ-isoform, albeit with large differences in total tocopherol contents and isoform profiles depending on cultivar.

Several pigments, including carotenoids and chlorophylls, are responsible for the cultivar-specific mango fruit pulp and peel color changes occurring during ripening. Most of them also show biological activities, acting as antioxidants or provitamins. Our results revealed differences in the carotenoid profile among the assayed mango fruit fractions. Peel samples had the highest total carotenoid content (1.75 mg/100 g dw in Tommy Atkins and 3.76 mg/100 g dw in Keitt), followed by pulp (0.71 and 1.22 mg/100 g dw, respectively) (Table 2). The cultivar Keitt exhibited significantly higher total carotenoid contents than Tommy Atkins, while kernels recorded similar contents. Seven carotenoids (four carotenes and three xanthophylls) were comprehensively identified. Violaxanthin was characteristic of the peel of the cultivar Keitt, lutein was not detected in the pulp of Tommy Atkins, and α-carotene characterized the peel of both cultivars. β-Carotene was detected in all fruit fractions and contributed about 80% and 86% to the total carotenoids of Tommy Atkins and Keitt pulp, respectively. β-carotene and violaxanthin were previously reported to prevail over the other carotenoids in Keitt and Tommy Atkins mangoes, accounting for 28–38% and 27–33% of the total, respectively, according to Mercandante et al. [73,74] and Ruales et al. [46]. However, the levels of β-carotene found by Mercandante et al. [73,74] in the pulp of Tommy Atkins and Keitt cultivars (0.58 and 1.5 mg/100 g dw, respectively) were similar to those reported in this study. Our findings are in agreement with the results obtained by Fratianni et al. [75], who reported the presence of 9-cis and 13-cis-isomers of β-carotene in the pulp of Keitt mangoes, although the total amount (about 12 mg/100 g dw) was much higher. Total carotenoids were 2.5–3-fold more concentrated in peels than in pulp. In the peels of Tommy Atkins, β-carotene contributed about 46% of the total carotenoids, followed by lutein (39%). Similar results were reported by Marcillo-Parra [43] for peels isolated from Tommy Atkins, Haden, and Kent cultivars. On the contrary, Keitt had 68% lutein and 23% β-carotene with respect to the total identified carotenoids. Some authors found lutein as the main carotenoid of peels, followed by β-carotene and low amounts of other carotenoids [46,76]. The high concentration of carotenoids in the peel is probably related to the high exposure to sunlight that induces an increase in carotengenesis [77]. Albeit in low quantity, kernel contained exclusively lutein and β-carotene.

Chlorophylls (a and b) were found in the peels of Tommy Atkins and Keitt cultivars (Table 2) in ratios of 1:3 and 1:4, respectively. Similar results were obtained by Dorta et al. [41] in freeze-dried peels of the cultivar Keitt.

### 3.4. Antioxidant Properties

Table 3 reports the HAA, LAA, and TAA of the pulp, peels and kernels obtained from the ripe fruits of both mango cultivars. HAA largely exceeded LAA in all fruit fractions, with both varying significantly among cultivars. Kernels showed the highest HAA (approximately 120 mol TE/100 g dw in both cultivars) and LAA (28.6 and 19.7 mol TE/100 g dw in Tommy Atkins and Keitt, respectively). After kernels, peels exhibited greater HAA and LAA than pulp. In particular, HAA and LAA of Keitt peel were greater than those evaluated in the peel of Tommy Atkins. Finally, the antioxidant activities determined in the pulp of both cultivars did not show statistically significant differences.

Several authors have previously investigated and demonstrated the positive correlation between TPC and antioxidant activity [78,79]. Our results (Table 4) indicated that TPC has a statistically significant (*p* < 0.001) positive Pearson correlation with HAA (*r* = 0.990). The strong correlation suggests that phenolic compounds present in the different mango fruit fractions likely contribute to radical scavenging activity, as also reported by Ma et al. [80]. Similarly, Puravankara et al. [81] found phenolic compounds as the main contributors to the HAA of mango seed kernel extracts, which, in line with the high phenolic content, exerted the highest antioxidant activity among several fruit seeds, including jackfruit, longan, avocado, and tamarind [82]. A strong correlation was also observed between HAA and TFC (*r* = 0.994). In previous studies, Rumainum et al. [78] reported a strong correlation between TFC and total antioxidant activity in Thai mangoes. Furthermore, HAA and LAA were positively correlated with TCT (*r* = 0.984, *r* = 0.982, respectively). Overall, our findings suggest that total phenolics, flavonoids, and condensed tannins are the major compounds contributing to the antioxidant activity in our samples. None of the lipophilic molecules correlates significantly with LAA, indicating an idiosyncratic rather than synergistic interaction of the individual contributors.

### 3.5. Principal Component Analysis

**Figure 5:**
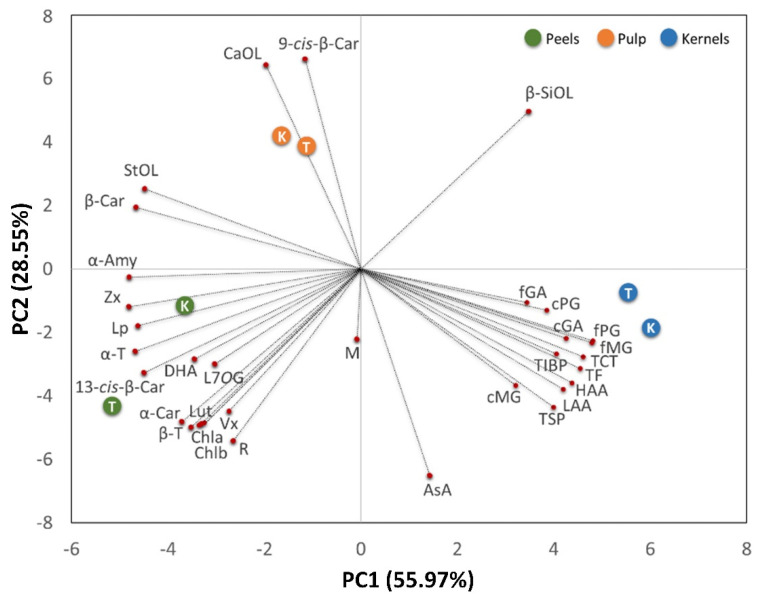
Principal component analysis (PCA) biplot PC1 vs. PC2 of total soluble phenolics (TSP), total insoluble bound phenolics (TIBP), total flavonoids (TF), total condensed tannins (TCT), free gallic acid (fGA), free methyl gallate (fMG), free propyl gallate (fPG), conjugated gallic acid (cGA), conjugated methyl gallate (cMG), conjugated propyl gallate (cPG), mangiferin (M), rutin (R), luteolin-7-*O*-glucoside (L7OG), ascorbic acid (AsA), dehydroascorbic acid (DHA), campesterol (CaOL), stigmasterol (StOL), β-sitosterol (β-SiOL), lupeol (Lp), α-amyrin (α-Amy), α-tocopherol (α-T), β-tocopherol (β-T), violaxanthin (Vx), lutein (Lut), zeaxanthin (Zx), α-carotene (α-Car), β-carotene (β-Car), 9-cis-β-carotene (9-cis-β-Car), 13-cis-β-carotene (13-cis-β-Car), chlorophyll a (Chla), chlorophyll b (Chlb), hydrophilic antioxidant activity (HAA) and lipophilic antioxidant activity (LAA) of different fruit tissues (pulp, peels and kernels) isolated from the ripe fruits of Tommy Atkins (T) and Keitt (K) mango cultivars. The variance (%) explained by each PCA axis is given in brackets. The length of the vectors is correlated to their significance within each population. Between vectors and between a vector and an axis, there is a positive correlation if the angle is <90°, whereas the correlation is negative if the angle reaches 180°. There is no linear dependence if the angle is 90°.

To highlight the biochemical differences among fruit fractions and cultivars and any clustering of the observations, a multivariate analysis (PCA) was carried out (Figure 5).

Two relevant principal components (PCs) that explained 84.52% of the total variance of the collected data were extracted, with principal component 1 (PC1) and principal component 2 (PC2) accounting for 55.97% and 28.55%, respectively. The contribution of each quality parameter (variables) is reported in Table 5. The PC1 vs. PC2 plot shows a clear clustering of fruit fractions.

Independently of cultivars, pulp fractions grouped on the upper-left side of the chart show a high concentration of phytosterols (mainly campesterol and β-sitosterol) and β- and 9-cis-β-carotene-isoforms. Phytosterols are lipophilic membrane components that are not only essential for diverse cellular functions, but are also biosynthetic precursors of the brassinosteroids [83]. Thus, their abundance in the mango pulp may be related to the extensive membrane system of the metabolically active parenchymal cells of the mesocarp tissue and/or to the physiological role of brassinosteroid phytohormones in the regulation of fleshy fruit ripening.

Peels grouped on the lower-left side panel, characterized by the presence of chlorophylls, responsible for the green colour, carotenes (α- and 13-cis-β-isoforms), and xanthophylls, are likely involved in photosynthesis as accessory pigments. Peels also correlated with lupeol, α-amyrin, and α- and β-tocopherols, presumably located in the cuticular waxes coating the surface of fleshy fruits with the role of limiting water loss, providing mechanical support, preventing fruit softening, and acting as a barrier to pathogens [84,85], as well as with dehydroascorbic acid, luteolin-7-*O*-glucoside, and rutin, which reflect the protective role that peel flavonoid compounds exert against the adverse biotic (e.g., pathogens, insects and herbivores attack) and abiotic (e.g., UV radiation and temperature) factors [86].

Kernels were distributed on the bottom right panel showing a high content phenolic (both soluble and insoluble), total flavonoid, total condensed tannins, as well as high hydrophilic and lipophilic antioxidant activities. As reported by Corso et al. [87] phenolics favor seed survival and dispersion and provide chemical defense against pathogens, seed defense against biotic attacks, as well as predators, such as insect pests and herbivores.

### 3.6. Fatty Acids Composition

The fatty acid profiles of the pulp, peels, and kernels obtained from the ripe fruits of Tommy Atkins and Keitt mango cultivars are reported in Table 6. Myristic, palmitic, stearic, arachidic, oleic, and linolenic acids were detected in all samples, though with significant quantitative differences. Palmitic was the most abundant fatty acid in the pulp (contributing to 18.5% and 20.1% of the total identified fatty acids in Tommy Atkins and Keitt, respectively), followed by the polyunsaturated fatty acids (PUFA) linolenic (15.9–19.8%) and linoleic (14.2–17.2%). The latter two, together with palmitic acid, prevailed in the peels of both cultivars, constituting up to 71.9% of the total. Myristic, heptadecanoic, stearic, arachidic, behenic, lignoceric, palmitoleic, and oleic acids were also detected as minor components in peels. Kernels were a rich source of oleic (38.4–41.6%), stearic (32.8–36.3%) and palmitic acids (13.8–11.1%). Our results fall within the ranges reported for the pulp, peels, and kernels of ripe mango fruits by other authors [65,88,89,90]. It is worth mentioning that some PUFA, including linoleic and linolenic acids, are essential nutrients for humans and must be introduced with the diet [89]. The role of n-3 and n-6 fatty acids for health is related with the prevention, delay, or treatment of chronic and acute diseases, including cancer, cardiovascular diseases, osteoporosis, and immune disorders [91,92,93,94]. Our analysis revealed an n6/n3 ratio <1, indicating mango fruits as a perfect source of essential fatty acids [88].

## 4. Conclusions

To the best of our knowledge, this is the first time that an in-depth characterization of several classes of bioactive molecules has been simultaneously performed in different fruit fractions of two mango cultivars (Tommy Atkins and Keitt).

Our findings demonstrate that although the pulp, peels, and kernels of ripe mango fruits are all interesting sources of bioactive molecules, significant variations in the qualitative and quantitative compositions were observed among them. In summary, independently of cultivars, pulp fractions had a high content of phytosterols and β- and 9-cis-β-carotene-isoforms. Peel fractions, instead, were characterized by the high concentration of chlorophylls, α- and 13-cis-β-carotene, xanthophylls, lupeol, α-amyrin, α- and β-tocopherols, dehydroascorbic acid, luteolin-7-*O*-glucoside, and rutin. Meanwhile, kernel fractions are an excellent reservoir of different classes of phenolic compounds exerting strong antioxidant activity. Furthermore, the highest percentage of SFA was presented in pulp and kernels, MUFA in kernels, and PUFA in peels.

The huge amount of agro-waste generated by food processing industries illustrates the rate of loss of valuable materials that could be used to produce useful products. Regrettably, the waste is not only discarded and unexploited, but poses serious environmental, economic, and management challenges. At present, technologies and inventions should seek to address zero waste and minimal pollution to the environment. This can only be achieved by using the various agro-industrial by-products as input to processes for the generation of new and value-added products.

Overall, the expansion of knowledge about the bioactive compounds in pulp, peels, and kernels supports the consumption of the mango as a healthy fruit and provides added value to peel- and kernel-based by-products, which can be used as potential novel ingredients to enhance the nutritional, antioxidant, and health-promoting properties for the development of new products in the food industry, as well as for the formulation for pharmaceutical and cosmeceutical purposes.

In addition, our findings suggest that the investigated agri-food by-products can be used as a source of valuable fatty acids. This appeals for additional research on the extraction, isolation, and purification of fatty acids from mango peels and kernels meeting the real market requirements.

Furthermore, it is worth considering that mango fruit contains allergenic substances, such as urushiol and resorcinols. Although rare cases of mango pulp-induced allergic contact dermatitis have been reported, a spectrum of hypersensitivity reactions has been associated to direct contact with peels particularly rich in these compounds, especially in subjects previously exposed to the urushiol containing plants, e.g., poison ivy (*Toxicodendron radicans* (L.) Kuntze) and poison oak (*T. diversilobum* (Torr. & A.Gray) Greene) [95,96]. Hence, the exploitation of mango peels must take into account the presence of these sensitizing substances in order to select an appropriate extraction method for obtaining a product free from potential allergens.

## Figures and Tables

**Table 1:** Quali-quantitative evaluation of the main soluble-free and soluble-conjugated phenols in the pulp, peels and kernels of ripe mango fruits of the cultivars Tommy Atkins and Keitt.

Soluble-Phenols	Fruit Fractions					
	Pulp		Peels		Kernels	
	mg/100 g dw					
	Tommy Atkins	Keitt	Tommy Atkins	Keitt	Tommy Atkins	Keitt
Free						
Gallic acid	nd	nd	nd	nd	**14.6 ± 0.9**	**104.2 ± 7.5**
Methyl gallate	**6.6 ± 0.2 ^C^**	**9.3 ± 0.2 ^C^**	**31.9 ± 1.9 ^B^**	**62.9 ± 0.1 ^B^**	**2126.5 ± 130.7 ^A^**	**1885.2 ± 66.6 ^A^**
Propyl gallate	nd	nd	**27.3 ± 0.4 ^B^**	**101.1 ± 0.9 ^B^**	1420.8 ± 63.5 ^A^	1375.2 ± 17.5 ^A^
*Total*	**6.6 ± 0.2 ^C^**	**9.3 ± 0.2 ^C^**	***59.2 ± 2.3 ^B^***	***164.0 ± 1.0 ^B^***	***3561.9 ± 195.1 ^A^***	***3364.6 ± 91.6 ^A^***
Conjugated						
Gallic acid	**197.1 ± 5.0 ^C^**	**189.2 ± 1.6 ^B^**	**129.7 ± 1.7 ^B^**	**214.6 ± 3.9 ^C^**	**851.3 ± 3.1 ^A^**	**338.2 ± 6.5 ^A^**
Methyl gallate	**23.6 ± 0.1 ^C^**	**3.8 ± 0.1 ^C^**	**367.7 ± 23.3 ^B^**	**559.4 ± 3.5 ^A^**	**387.4 ± 35.9 ^A^**	**nd**
Propyl gallate	nd	nd	nd	nd	nd	nd
Mangiferin	nd	nd	**411.0 ± 2.4 ^A^**	nd	**185.8 ± 1.7 ^B^**	**57.9 ± 0.2**
Rutin	nd	nd	**65.2 ± 1.6 ^A^**	**30.2 ± 0.3 ^A^**	**40.3 ± 0.1 ^B^**	**25.5 ± 6.1 ^B^**
Luteolin 7-*O*-glucoside	nd	nd	**13.1 ± 0.1**	**5.3 ± 0.3**	nd	nd
*Total*	**220.7 ± 5.1 ^C^**	**193.0 ± 1.7 ^C^**	**986.7 ± 29.1 ^B^**	**809.5 ± 8.0 ^B^**	**1464.8 ± 40.8 ^A^**	**421.6 ± 12.8 ^A^**

nd: not detected. Data, expressed as mg/100 g dw, are the mean ± standard deviation of five independent replicates (*n* = 5). Data were submitted to one-way analysis of variance (ANOVA), differences among groups were detected using multiple comparison procedures (Tukey test). Bold indicates statistically significant differences between cultivars within the same fruit fraction; different capital letters denote differences among fruit fractions within the same cultivar (*p* < 0.05).

**Table 2:** Quali-quantitative evaluation of the main lipophilic phytochemicals (phytosterols, pentacyclic triterpenes, tocopherols, carotenoids and chlorophylls) in the pulp, peels and kernels of ripe mango fruits of the cultivars Tommy Atkins and Keitt.

	Fruit Fractions					
	Pulp		Peels		Kernels	
	mg/100 g dw					
	Tommy Atkins	Keitt	Tommy Atkins	Keitt	Tommy Atkins	Keitt
Phytosterols						
Campesterol	11.4 ±0.2 ^A^	11.5 ± 0.8 ^A^	**3.0 ± 0.2 ^B^**	**1.4 ± 0.1 ^B^**	nd	nd
Stigmasterol	**11.8 ± 0.5 ^B^**	**20.8 ± 0.2 ^A^**	13.9 ± 1.6 ^A^	15.3 ± 1.4 ^B^	nd	nd
ß-sitosterol	128.4 ± 16.2 ^A^	117.6 ± 19.2 ^A^	**66.2 ± 0.6 ^B^**	**39.2 ± 3.6 ^B^**	**106.9 ± 7.6 ^A^**	**135.5 ± 18.2 ^A^**
*Total*	151.5 ± 16.9 ^A^	150.0 ± 20.1 ^A^	**83.0 ± 2.4 ^B^**	**55.9 ± 5.1 ^B^**	**106.9 ± 7.6 ^B^**	**135.5 ± 18.2 ^A^**
Pentacyclic triterpenes						
Lupeol	**2.3 ± 0.3 ^A^**	**4.6 ± 0.2 ^B^**	**9.3 ± 0.4 ^B^**	**7.9 ± 0.4 ^A^**	nd	nd
α -amyrin	**4.0 ± 0.2 ^B^**	**4.6 ± 0.3 ^B^**	**8.4 ± 0.3 ^A^**	**6.3 ± 0.6 ^A^**	nd	nd
*Total*	**6.3 ± 0.4 ^B^**	**9.1 ± 0.5 ^B^**	**17.7 ± 0.7 ^A^**	**14.2 ± 1.3 ^A^**	nd	nd
Tocopherols						
α -tocopherol	**2.3 ± 0.3 ^C^**	**3.7 ± 0.1 ^B^**	**7.5 ± 0.3 ^A^**	**8.7 ± 0.3 ^A^**	0.2 ± 0.1 ^B^	0.1 ± 0.1 ^C^
ß-tocopherols	nd	nd	**3.0 ± 0.2 ^A^**	**3.6 ± 0.4 ^A^**	0.3 ± 0.1 ^B^	0.1 ± 0.1 ^B^
*Total*	**2.3 ± 0.3 ^B^**	**3.7 ± 0.1 ^B^**	**10.6 ± 0.5 ^A^**	**12.3 ± 0.8 ^A^**	**0.5 ± 0.2 ^C^**	**0.2 ± 0.2 ^C^**
Carotenoids						
Violaxanthin	nd	nd	nd	0.04 ± 0.00	nd	nd
Lutein	**0.02 ± 0.01 ^B^**	**nd**	**0.69 ± 0.03 ^A^**	**2.56 ± 0.11 ^A^**	0.01 ± 0.00 ^B^	0.01 ± 0.00 ^B^
Zeaxanthin	0.03 ± 0.01 ^A^	0.02 ± 0.01 ^B^	**0.03 ± 0.01 ^A^**	**0.05 ± 0.01 ^A^**	nd	nd
α-carotene	nd	nd	**0.08 ± 0.00**	**0.11 ± 0.00**	nd	nd
β-carotene	**0.60 ± 0.10 ^B^**	**1.08 ± 0.03 ^A^**	0.83 ± 0.07 ^A^	0.88 ± 0.08 ^B^	0.01 ± 0.00 ^C^	0.01 ± 0.00 ^C^
9-cis-β-carotene	0.07 ± 0.01	0.07 ± 0.01	nd	nd	nd	nd
13-cis-β-carotene	**0.03 ± 0.01 ^B^**	**0.05 ± 0.00 ^B^**	**0.12 ± 0.01 ^A^**	**0.16 ± 0.01 ^A^**	nd	nd
*Total*	**0.71 ± 0.14 ^B^**	**1.22 ± 0.05 ^B^**	**1.75 ± 0.11 ^A^**	**3.76 ± 0.20 ^A^**	0.02 ± 0.00 ^C^	0.01 ± 0.00 ^C^
Chlorophylls						
Chlorophyll a	nd	nd	**0.48 ± 0.03**	**1.59 ± 0.01**	nd	nd
Chlorophyll b	nd	nd	**1.39 ± 0.02**	**6.01 ± 0.22**	nd	nd
*Total*	nd	nd	**1.87 ± 0.05**	**7.60 ± 0.23**	nd	nd

nd, not detected. Data, expressed as mg/100 g dw, are the mean ± standard deviation of five independent replicates (*n* = 5). Data were submitted to one-way analysis of variance (ANOVA), differences among groups were detected using multiple comparison procedures (Tukey test). Bold indicates statistically significant differences between cultivars within the same fruit fraction; different capital letters denote differences among fruit fractions within the same cultivar (*p* < 0.05).

**Table 3:** Hydrophilic (HAA), lipophilic (LAA) and total (TAA) antioxidant activities in the pulp, peels and kernels of ripe mango fruits of the cultivars Tommy Atkins and Keitt.

	Fruit Fractions					
	Pulp		Peels		Kernels	
	mM TE/g dw					
	Tommy Atkins	Keitt	Tommy Atkins	Keitt	Tommy Atkins	Keitt
HAA	5.1 ± 0.1 ^C^	5.1 ± 0.1 ^C^	**24.9 ± 0.8 ^B^**	**35.9 ± 3.7 ^B^**	122.4 ± 0.5 ^A^	116.2 ± 9.8 ^A^
LAA	2.7 ± 0.3 ^C^	2.5 ± 0.2 ^C^	**6.2 ± 0.4 ^B^**	**9.2 ± 1.0 ^B^**	**28.6 ± 1.6 ^A^**	**19.7 ± 1.6 ^A^**
TAA	7.8 ± 0.4 ^C^	7.6 ± 0.3 ^B^	**31.1 ± 1.2 ^B^**	**45.1 ± 4.7 ^B^**	151.0 ± 2.1 ^A^	135.9 ± 11.4 ^A^

Data, expressed as mM Trolox equivalents (TE)/g dw, are mean ± standard deviation of five independent replicates (*n* = 5). Data were submitted to one-way analysis of variance (ANOVA), differences among groups were detected using multiple comparison procedures (Tukey test). Bold indicate statistically significant differences between cultivars within the same fruit fraction; different capital letters denote differences among fruit fractions within the same cultivar (*p* < 0.05).

**Table 4:** Pearson’s correlation for antioxidant activities (TEAC method) versus antioxidant compounds. *n* (sample size) = 5. Values in bold are significant.

Antioxidants	HAA		LAA	
	r	*p*	r	*p*
TPC	**0.990**	<0.001	0.915	0.01
TFC	**0.994**	<0.0001	0.958	0.002
TCT	**0.984**	<0.001	**0.982**	<0.001
AsA	0.703	0.119	0.620	0.190
DHA	−0.396	0.437	−0.401	0.431
TP	−0.141	0.789	−0.017	0.974
TPT	−0.723	0.104	−0.749	0.086
TT	−0.500	0.312	−0.555	0.253
TC	−0.511	0.300	−0.553	0.255
TCh	−0.207	0.694	−0.264	0.613

HAA, hydrophilic antioxidant activity; LAA, lipophilic antioxidant activity; r, Pearson’s correlation coefficient; *p*, *p*-value; TPC, total phenolic compounds; TFC, total flavonoid compounds; TCT, total condensed tannins; AsA, ascorbic acid; DHA, dehydroascorbic acid; TP, total phytosterols; TPT, total pentacyclic triterpenes; TT, total tocopherols; TC, total carotenoids; TCh, total chlorophylls.

**Table 5:** Variables participating in the construction of the factorial axes and their relative contribution (%) to PCA dimensions.

Variables	Contribution (%)	
	PC1	PC2
TSP	3.350	3.983
TIBP	3.446	1.491
TF	4.334	2.059
TCT	4.447	1.610
fGA	2.483	0.232
fMG	4.855	1.074
fPG	4.807	1.134
cGA	3.787	1.004
cMG	2.160	2.810
cPG	3.110	0.355
M	0.001	1.027
R	1.462	6.131
L7OG	1.915	1.874
AsA	0.428	8.860
DHA	2.489	1.692
CaOL	0.807	8.669
StOL	4.206	1.337
β-SiOL	2.538	5.162
Lp	4.475	0.672
α-Amy	4.828	0.014
α-T	4.575	1.408
β-T	2.594	5.203
Vx	1.558	4.206
Lut	2.289	4.993
Zx	4.841	0.295
α-Car	2.886	4.846
β-Car	4.553	0.791
9-*cis*-β-Car	0.280	9.156
13-*cis*-β-Car	4.232	2.232
Chla	2.364	5.059
Chlb	2.195	4.933
HAA	4.020	2.691
LAA	3.685	2.998

TSP, total soluble phenolics; TIBP, total insoluble bound phenolics; TF, total flavonoids; TCT, total condensed tannins; fGA, free gallic acid; fMG, free methyl gallate; fPG, free propyl gallate; cGA conjugated gallic acid; cMG, conjugated methyl gallate; cPG, conjugated propyl gallate; M, mangiferin; R, rutin; L7OG, luteolin-7-*O*-glucoside; AsA, ascorbic acid; DHA, dehydroascorbic acid; CaOL, campesterol; StOL, stigmasterol; β-SiOL, β-sitosterol; Lp, lupeol; α-Amy, α-amirin; α-T, α-tocopherol; β-T, β-tocopherol; Vx, violaxanthin; Lut, lutein; Zx, zeaxanthin; α-Car, α-carotene; β-Car, β-carotene; 9-cis-β-Car, 9-cis-β-carotene; 13-cis-β-Car, 13-cis-β-carotene; Chla, chlorophyll a; Chlb, chlorophyll b; HAA hydrophilic antioxidant activity; LAA lipophilic antioxidant activity.

**Table 6:** Fatty acids composition of three fruit fractions of mango fruit (pulp, peels and kernels).

Fatty Acids	Fruit Fractions					
	Pulp		Peels		Kernels	
	% of Total Identified Fatty Acids					
	Tommy Atkins	Keitt	Tommy Atkins	Keitt	Tommy Atkins	Keitt
Myristic acid (C14:0)	**4.8** **±** **0.7 ^A^**	**3.2** **±** **0.4 ^A^**	**5.4** **±** **0.9 ^A^**	**3.4** **±** **0.4 ^A^**	nd	nd
Palmitic acid (C16:0)	**18.5** **±** **2.4 ^AB^**	**20.1** **±** **2.7 ^A^**	20.8 ± 2.7 ^A^	19.3 ± 2.7 ^A^	**13.8** **±** **1.3 ^B^**	**11.1** **±** **1.1 ^B^**
Heptadecanoic acid (C17:0)	**1.1** **±** **0.1 ^A^**	**0.9** **±** **0.1 ^A^**	0.6 ± 0.1 ^B^	0.7 ± 0.1 ^A^	nd	nd
Stearic acid (C18:0)	17.2 ± 2.3 ^C^	17.1 ± 2.2 ^B^	**6.8** **±** **0.8 ^B^**	**9.4** **±** **1.2 ^C^**	**32.8** **±** **3.0 ^A^**	**37.3** **±** **3.1 ^A^**
Arachidic acid (C20:0)	**1.1** **±** **0.1 ^B^**	**0.9** **±** **0.1 ^B^**	1.2 ± 0.2 ^B^	1.3 ± 0.2 ^B^	**2.5** **±** **0.2 ^A^**	**1.8** **±** **0.1 ^A^**
Behenic acid (C22:0)	nd	nd	1.4 ± 0.3	1.2 ± 0.2	nd	nd
Lignoceric acid (C24:0)	nd	nd	0.9 ± 0.1	0.9 ± 0.1	nd	nd
Palmitoleic acid (C16:1)	8.2 ± 1.1 ^A^	5.7 ± 0.7 ^A^	**2.7** **±** **0.4 ^B^**	**2.1** **±** **0.3 ^B^**	nd	nd
Oleic acid (C18:1n-9c)	**10.2** **±** **1.3 ^B^**	**7.2** **±** **1.0 ^B^**	**9.5** **±** **1.2 ^B^**	**6.7** **±** **0.8 ^B^**	**38.4** **±** **3.7 ^A^**	**41.5** **±** **3.9 ^A^**
11-octadecenoic acid (C18:1n-7c)	8.8 ± 1.2 ^A^	8.0 ± 1.1 ^A^	**3.3** **±** **0.4 ^B^**	**2.6** **±** **0.3 ^B^**	nd	nd
Linoleic acid (C18:2n-6)	**14.3** **±** **1.7 ^B^**	**17.2** **±** **1.9 ^B^**	**25.8** **±** **3.1 ^A^**	**29.9** **±** **3.9 ^A^**	**11.0** **±** **1.0 ^C^**	**7.6** **±** **0.7 ^C^**
Linolenic acid (C18:3n-3)	**15.9** **±** **1.7 ^B^**	**19.8** **±** **2.6 ^B^**	**21.6** **±** **2.7 ^A^**	**22.5** **±** **2.7 ^A^**	**1.5** **±** **0.1 ^C^**	**0.6** **±** **0.06 ^C^**
SFA	42.7 ± 5.6 ^AB^	42.1 ± 5.5 ^AB^	37.1 ± 5.1 ^B^	36.2 ± 4.9 ^B^	49.1 ± 4.5 ^A^	50.2 ± 4.3 ^A^
MUFA	**27.1** **±** **3.6 ^B^**	**20.9** **±** **2.8 ^B^**	**15.5** **±** **2.0 ^C^**	**11.4** **±** **1.4 ^C^**	38.4 ± 3.7 ^A^	41.5 ± 3.9 ^A^
PUFA	**30.2** **±** **3.4 ^B^**	**37.0** **±** **4.5 ^B^**	47.4 ± 5.8 ^A^	52.4 ± 6.6 ^A^	**12.5** **±** **1.1 ^C^**	**8.2** **± 0.76 ^C^**

nd, not detected. Data, expressed on a percentage basis, are the mean ± standard deviation of three independent replicates (*n* = 3). Data were submitted to one-way analysis of variance (ANOVA), differences among groups were detected using multiple comparison procedures (Tukey test), values in bold denote significant differences between the cultivar within the same fruit fraction; capital letters indicate differences among different fraction for the same cultivar (*p* < 0.05).

## Data Availability

Data is contained within the article.
